# Delayed traumatic diaphragmatic hernia: A case-series report and literature review

**DOI:** 10.1097/MD.0000000000004362

**Published:** 2016-08-12

**Authors:** Jing Lu, Bo Wang, Xiangming Che, Xuqi Li, Guanglin Qiu, Shicai He, Lin Fan

**Affiliations:** a Department of General Surgery; b Department of Hepatobiliary Surgery, the First Affiliated Hospital, School of Medicine, Xi’an Jiaotong University, Xi’an Shaanxi, P.R. China.

**Keywords:** diaphragmatic injury, hernia, trauma

## Abstract

**Background:**

Traumatic diaphragmatic hernias (TDHs) are sometimes difficult to identify at an early stage and can consequently result in diagnostic delays with life-threatening outcomes. It is the aim of this case study to highlight the difficulties encountered with the earlier detection of traumatic diaphragmatic hernias.

**Methods:**

Clinical data of patients who received treatment for delayed traumatic diaphragmatic hernias in registers of the First Affiliated Hospital of Xi’an Jiaotong University from 1998 to 2014 were analyzed retrospectively.

**Results:**

Six patients were included in this study. Left hemidiaphragm was affected in all of them. Most of the patients had a history of traffic accident and 1 a stab-penetrating injury. The interval from injury to developing symptoms ranged from 2 to 11 years (median 5 years). The hernial contents included the stomach, omentum, small intestine, and colon. Diaphragmatic injury was missed in all of them during the initial managements. All patients received operations once the diagnosis of delayed TDH was confirmed, and no postoperative mortality was detected.

**Conclusions:**

Delayed TDHs are not common, but can lead to serious consequences once occurred. Early detection of diaphragmatic injuries is crucial. Surgeons should maintain a high suspicion for injuries of the diaphragm in cases with abdominal or lower chest traumas, especially in the initial surgical explorations. We emphasize the need for radiographical follow-up to detect diaphragmatic injuries at an earlier stage.

## 1 Introduction

Traumatic diaphragmatic hernias (TDHs) are uncommon, but life-threatening, and remain a diagnostic and therapeutic challenge with an overall mortality rate of up to 31% in recent series. ^[1]^ Patients with traumatic diaphragmatic hernias often had a history of penetrating or blunt trauma. And they may stay unrecognized after injury, only presenting months or years later with strangulation of an incarcerated abdominal viscus and respiratory compromise owing to reduced intrathoracic volume. Therefore missed diagnosis may occur during the initial evaluation after the injury. In this retrospective study, we report on patients suffering from delayed TDH treated in our center, and we discuss the diagnosis and treatment of delayed TDHs.

## 2 Patients and methods

We performed a retrospective study and retrieved the medical records of all patients with delayed TDH in registers of the First Affiliated Hospital of Xi’an Jiaotong University from 1998 to 2014.

According to the classification originally proposed by Carter et al, ^[2]^ traumatic diaphragmatic hernias are grouped into 3 phases: acute phase (within 14 days), interval phase, and obstructive phase. The latter 2 phases are collectively referred to as delayed TDH. In this study, the diagnosis of delayed TDH was the single inclusion criteria to be fulfilled.

Under Chinese law, this type of study, which does not involve any invasive investigation, but relies on a retrospective analysis of patient files, does not need the approval of the institutional review board. Written informed consent was obtained from the patients for publication of this article and any accompanying images.

## 3 Results

### 3.1 General information

A total of 6 patients were finally included in the study (Table 1). All the patients were male, with a median age of 39 years (ranging from 29 to 51 years). The left hemidiaphragm was affected in all of them. Five of them had a history of traffic accidents. Three of these patients had suffered multiple rib fractures with splenic rupture who underwent splenectomy after the injury, 2 had hemopneumothorax and pelvic fracture, respectively, who received neither celiotomy nor thoracotomy at that time. One patient sustained injury secondary to a stab-penetrating trauma at the seventh intercostal space of the back 5 years earlier, and was treated conservatively. The interval from injury to the arising of overt symptoms ranged from 2 to 11 years (median 5 years). The hernial contents included the stomach and omentum in 4 cases, stomach and small intestine in 1 case, and colon in the other one.

**Table 1:**
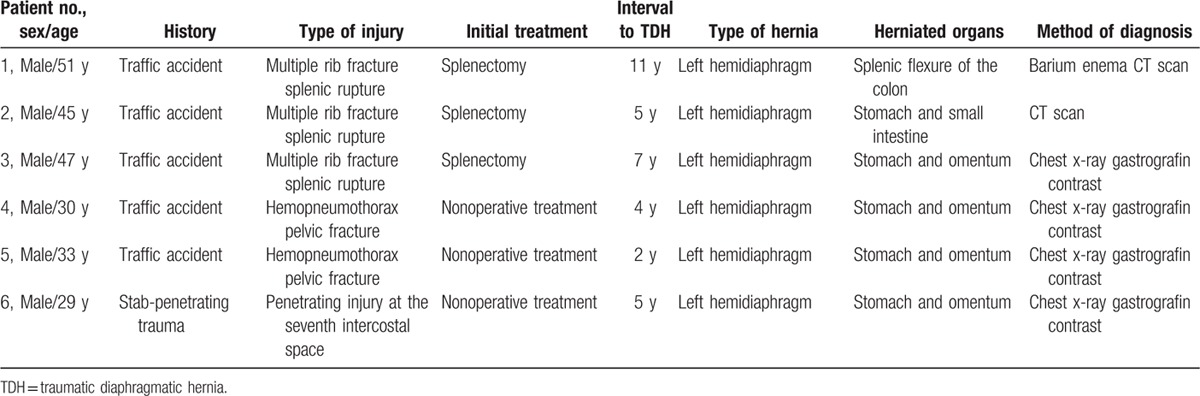
Summary of the 6 patients included in the study.

The common presentations of the patients included abdominal pain, nausea, vomiting, dyspnea, breathlessness, shoulder pain, or chest pain. Besides, recurrent abdominal distension and chest pain after a meal occurred in 2 patients. Physical examination revealed decreased breath sounds on the left side and deviation of the trachea to the right side. We noted bowel sounds in the left hemithorax in 2 of 6 patients. Chest x-ray showed left pleural effusion and left pulmonary collapse in all cases. Chest x-ray or computed tomography (CT) scan demonstrated herniation of stomach and/or bowel loops into the left thorax. All patients underwent operations once the diagnosis of delayed TDH was confirmed and went through an uneventful postoperative course. No prosthetic materials were required to cover the diaphragmatic defect. We noted no recurrence of a hernia by the latest follow-up. All the patients recovered well.

### 3.2 Specific findings and management

**Figure 1:**
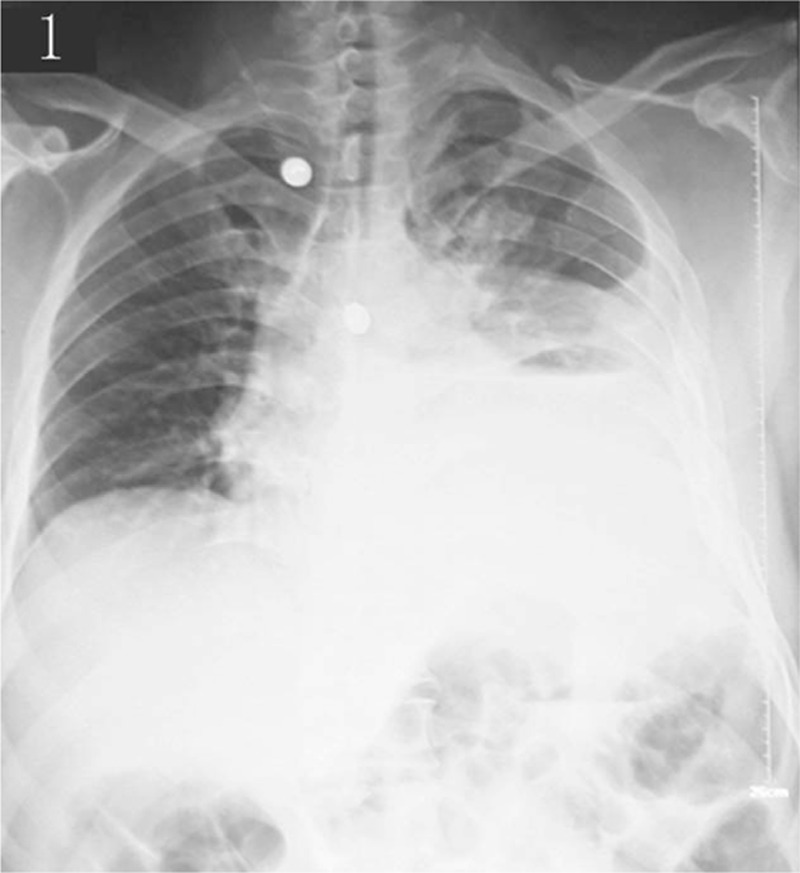
Chest x-ray revealing a left gastrothorax.

**Figure 2:**
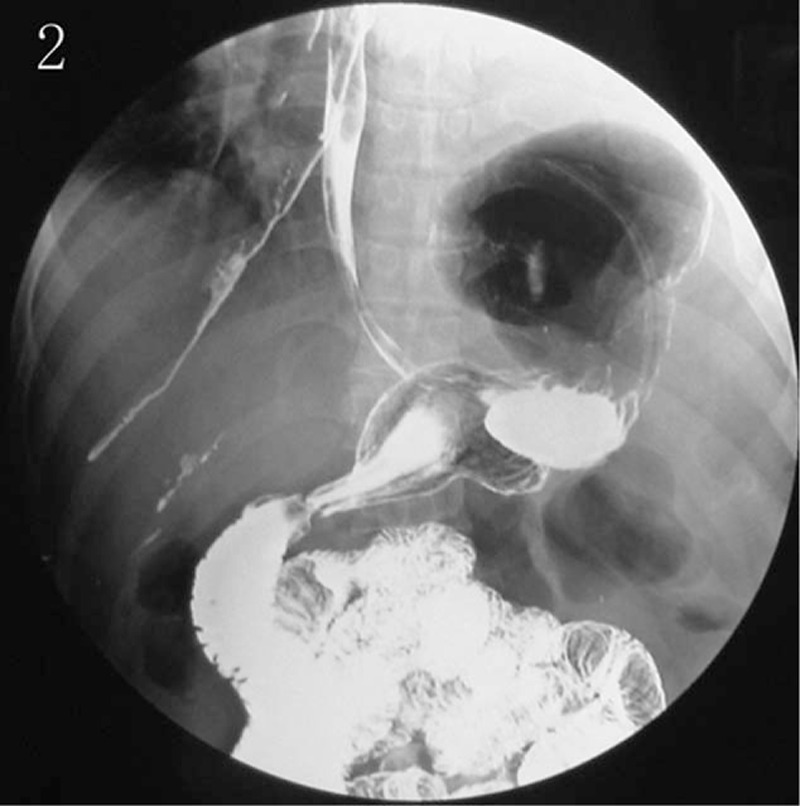
Alimentary tract contrast examination showing the herniation.

Of the 6 patients, 4 had chest x-ray and chest radiograph after administration of gastrografin with findings of stomach herniated into the left hemithorax (Figs. 1 and 2). We inserted a thoracic tube in one of these patients. The tube drained about 1 L of bloody fluid per day and the patient underwent emergency operation through an abdominal incision. We noted a 6 cm long tear in the left hemidiaphragm (stab-penetrating injury 5 years ago), with the whole stomach herniated into thorax and the pleural cavity full of bloody gastric fluid resulting in strangulation and necrosis of the proximal stomach. We undertook a partial resection of the stomach, and repaired the diaphragm with interrupted nonabsorbable sutures. The remaining 3 patients who suffered from a herniated stomach underwent thoracotomy, and we reduced the herniations of the stomach and omentum into the abdomen. Direct closure of the diaphragm using interrupted nonabsorbable sutures was performed.

**Figure 3:**
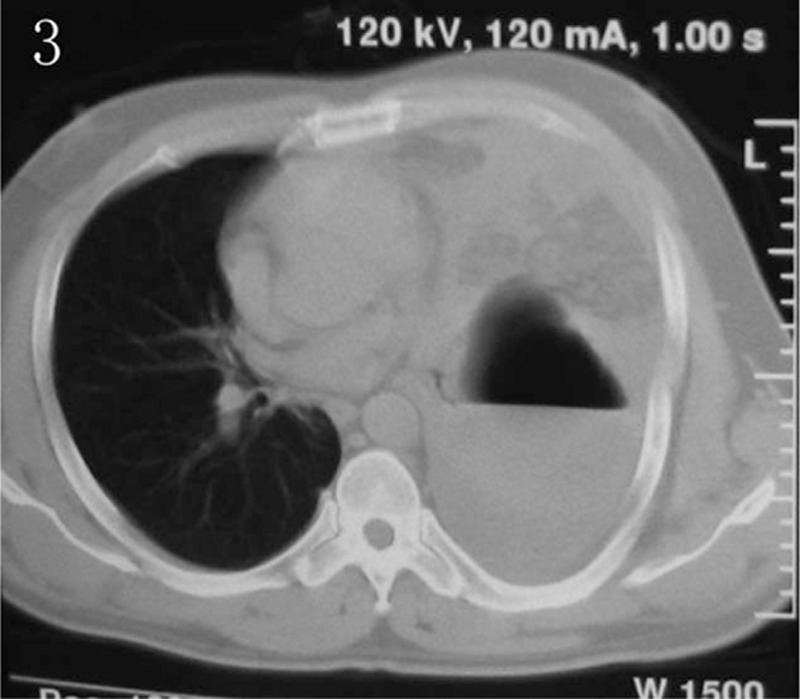
Computed tomography (CT) scan indicating the stomach and bowel loops herniated into the left thorax.

One of the 6 patients had a CT scan indicating stomach and bowel loops herniated through a left-sided diaphragmatic defect (Fig. 3). Thoracotomy was performed in the patient. And the hernia contents were found to be stomach and small intestine. The well perfused stomach and bowel were returned into the abdominal cavity and the diaphragm was repaired.

One patient has undergone splenectomy after involvement in a motor vehicle accident 11 years ago. He presented with a history of intermittent chest pain and mechanical ileus for 1 year. Diaphragmatic herniation of the colon was confirmed by barium enema and CT scan, which revealed a loop of the colon herniating into the left hemithorax. A laparotomy was performed in this patient. The splenic flexure of the colon and omentum were found entering into the thorax through a 7 cm long diaphragmatic defect. We reduced the herniated organs and repaired the diaphragmatic defect.

## 4 Discussion

Traumatic diaphragmatic hernias are thought to be produced by a sudden increase in the pleuroperitoneal pressure gradient, ^[3]^ occurring at areas of potential weakness along embryological points of fusion. TDHs usually result from blunt or penetrating injuries or iatrogenic causes, and result in entry of an abdominal hollow viscus or the omentum into the pleural cavity, which may lead to incarceration and even strangulation with a fatal outcome. ^[4]^ The most common cause of TDH is blunt thoracoabdominal trauma, such as road traffic accident and fall from height. It occurs in approximately 3% of abdominal injuries with a 2:1 ratio of penetrating to blunt trauma. ^[5]^ In this study, 5 of the patients had a history of traffic accidents, which accounted for 83% of the cases. Only 1 patient had a stab-penetrating injury.

The left hemidiaphragm is more commonly involved in blunt or penetrating injuries. ^[6]^ In our patients, the left hemidiaphragm was injured in all the patients. It was reported that this is probably due to the protective effect of the liver for the right hemidiaphragm in blunt trauma, and the fact that most people use their right hand for protection against penetrating trauma. ^[7]^

In penetrating traumas, there is no predisposing area of injury and the defect of the diaphragm is usually smaller than the defects caused by blunt traumas. Therefore, TDHs caused by penetrating injuries are potentially more dangerous in terms of possible obstruction and strangulation of the intestine. ^[8]^ In our study, the one who had suffered a penetrating injury before presented with strangulation and necrosis of the proximal part of the stomach.

Reber et al ^[8]^ reported that the rate of initially missed diaphragmatic injuries during the treatment of combined thoracoabdominal injuries can be as high as 66%. Even in patients undergoing laparotomy, there is still a risk of overlooking the diaphragmatic defect. Miller et al ^[9]^ reported that in a series of 93 patients with penetrating injuries of the diaphragm, the radiographs were interpreted as normal in 40 of them. Surgeons are paying closer attention to the lethal injuries (splenic rupture, liver damage, etc) during the emergency operation. To omit a thorough exploration of the entire abdomen may result in missed detection of diaphragm injury. In 3 of our cases, who had suffered a splenic rupture during traffic accidents and received splenectomy immediately years ago, the TDHs went unrecognized and thus were not repaired. Three of 6 patients who either had pelvic fracture and hemopneumothorax from traffic accidents or had stab-penetrating injury were all treated nonoperatively due to the lacking of indications of diaphragm injury in radiographic examinations at that time.

Traumatic diaphragmatic injuries are difficult to diagnose in emergency settings unless it is already accompanied by herniation of intra-abdominal contents. We noted that conventional plain x-ray, CT scan, or ultrasonography proved not useful in determining an acute diaphragmatic injury, although they represent beneficial methods in detecting the diaphragmatic hernia when there was established herniation. However, the data from the study by Sarita et al ^[10]^ and our recent data show that helical CT and multislice CT, such as 64-slice multidetector row spiral computed tomography (64-MDCT), may allow better demonstration of most subtle signs of diaphragmatic injury with high sensitivity, specificity, and accuracy because of a dramatic reduction in motion, beam-hardening artifacts, and significant improvement of spatial resolution. The usual features of diaphragmatic injury seen on MDCT scan are diaphragmatic discontinuity, thickened diaphragm, “collar sign,” visceral herniation and dependent viscera sign, and so on. For patients suffering from thoracoabdominal injuries who have not yet developed overt radiologic signs even with MDCT, diaphragmatic injury should be suspected in all patients and careful examination of the entire traumatized area during surgery is the best approach in preventing delayed TDH before development of severe complications. In addition, radiographical follow-ups should be undertaken in case diaphragmatic injuries are not definitely excluded during the first management. By accurate follow-up, we can detect diaphragmatic injuries to the greatest extent and at an earlier phase.

Once diagnosed, operation of delayed TDH (dTDH) is mandatory to reduce the risk of subsequent complications. There are many large series which describe the repair of dTDH by laparotomy, thoracotomy, laparoscopy, or thoracoscopy. [^11^ ^12^ ^13^] In this study, we performed operations both ways.

## 5 Conclusions

Delayed traumatic diaphragmatic hernias are not common, but can lead to serious consequences once occurred. Early detection of diaphragmatic injuries is crucial to prevent the occurrence of dTDHs. Surgeons should maintain a high suspicion for injuries of the diaphragm in patients who had suffered abdominal or lower chest traumas, especially during the initial surgical explorations. The need for radiographical follow-ups is emphasized to detect diaphragmatic injuries at an earlier stage.
